# The Same Microbiota and a Potentially Discriminant Metabolome in the Saliva of Omnivore, Ovo-Lacto-Vegetarian and Vegan Individuals

**DOI:** 10.1371/journal.pone.0112373

**Published:** 2014-11-05

**Authors:** Francesca De Filippis, Lucia Vannini, Antonietta La Storia, Luca Laghi, Paola Piombino, Giuseppina Stellato, Diana I. Serrazanetti, Giorgia Gozzi, Silvia Turroni, Ilario Ferrocino, Camilla Lazzi, Raffaella Di Cagno, Marco Gobbetti, Danilo Ercolini

**Affiliations:** 1 Department of Agricultural Sciences, Division of Microbiology, University of Naples Federico II, Portici, Italy; 2 Department of Agricultural and Food Sciences, Alma Mater Studiorum University of Bologna, Bologna, Italy; 3 Inter-Departmental Centre for Industrial Agri-Food Research, Alma Mater Studiorum University of Bologna, Cesena, Italy; 4 Department of Pharmacy and Biotechnology, Alma Mater Studiorum University of Bologna, Bologna, Italy; 5 Department of Agricultural, Forest and Food Science, University of Turin, Grugliasco, Italy; 6 Department of Food Science, University of Parma, Parma, Italy; 7 Department of Soil, Plant and Food Science, University of Bari Aldo Moro, Bari, Italy; Graz University of Technology (TU Graz), Austria

## Abstract

The salivary microbiota has been linked to both oral and non-oral diseases. Scant knowledge is available on the effect of environmental factors such as long-term dietary choices on the salivary microbiota and metabolome. This study analyzed the microbial diversity and metabolomic profiles of the saliva of 161 healthy individuals who followed an omnivore or ovo-lacto-vegetarian or vegan diet. A large core microbiota was identified, including 12 bacterial genera, found in >98% of the individuals. The subjects could be stratified into three “salivary types” that differed on the basis of the relative abundance of the core genera *Prevotella*, *Streptococcus/Gemella* and *Fusobacterium/Neisseria*. Statistical analysis indicated no effect of dietary habit on the salivary microbiota. Phylogenetic beta-diversity analysis consistently showed no differences between omnivore, ovo-lacto-vegetarian and vegan individuals. Metabolomic profiling of saliva using ^1^H-NMR and GC-MS/SPME identified diet-related biomarkers that enabled a significant discrimination between the 3 groups of individuals on the basis of their diet. Formate, urea, uridine and 5-methyl-3-hexanone could discriminate samples from omnivores, whereas 1-propanol, hexanoic acid and proline were characteristic of non-omnivore diets. Although the salivary metabolome can be discriminating for diet, the microbiota has a remarkable inter-individual stability and did not vary with dietary habits. Microbial homeostasis might be perturbed with sub-standard oral hygiene or other environmental factors, but there is no current indication that a choice of an omnivore, ovo-lacto-vegetarian or vegan diet can lead to a specific composition of the oral microbiota with consequences on the oral homeostasis.

## Introduction

The oral cavity is exposed to the external environment and is, therefore, one of the most important ways of microbial entry in the human body. Saliva plays a pivotal role in the maintenance of oral homeostasis and protection from disease [1]. The study of the oral microbiome is fundamental to understanding how and why oral microbial complexity can switch from a protective role to being a cause of disease [2]. Several studies have reported on the salivary microbiota as related to oral disease [3]–[6] and a gradual changing of the oral microbial community (dysbiosis) can progressively lead to a state of clinical disease [7]. Recently, a metatranscriptome study carried out on salivary microbiome revealed differences in microbial gene expression in case of oral disbyosis [8]. The recent literature also reports associations between the salivary microbiota and non-oral disease. Specific microbial consortia have been associated with obesity [9], [10], cancer [11], [12], HIV [13], inflammatory bowel disease [14], and atherosclerosis [15]. The possible value of the salivary microbiota for the early diagnosis of disease has also been noted [2]. A high degree of microbial diversity was reported for oral environments [16]–[18], and this level of diversity decreases in cases of oral diseases [2]. The association between high diversity and oral health suggests that each member of the complex microbial community has a function in maintaining homeostasis in the oral cavity. In this context, it is likely that a core microbiota in the saliva of healthy humans exists [19] and that it can play a protective role in the health of the oral environment.

Some important issues to address are the factors affecting the structure of the oral microbiota and understanding how the homeostasis of microbial complexity might be perturbed in a way leading to a predisposition to disease. To date, studies enrolling a significant number of individuals have demonstrated that the salivary microbiota does not vary markedly with geographic location [16], and is very stable, such that individuals maintain very similar microbiota over time [17], [20]. It was hypothesized that environmental factors such as dietary behavior and oral hygiene can have the most significant effect on the oral microbial composition [17]. Nevertheless, there are no studies linking oral microbiota to dietary habits.

Salivary metabolomics, mainly based on ^1^H nuclear magnetic resonance (^1^H-NMR) spectroscopy and mass spectrometry, have been used for the high-throughput identification of disease-associated salivary biomarkers and to facilitate the early diagnosis of several diseases [21], [22]. Salivary metabolomic profiles can be affected by both physiological and environmental factors [23].

Although it is well known that nutrition influences health and metabolism in many ways and a study on the role of dietary intervention in affecting dental health was recently carried out [24], comparatively little knowledge is available on the effect of dietary habits on the salivary microbiota and metabolome. The choice of following ovo-lacto-vegetarian or vegan diets is increasing worldwide due to the increasingly important benefits that such diets can have on human health, including possible prevention of cardio-vascular diseases (CVD), cancer and diabetes [25], [26]. However, it remains unclear whether such long-term dietary choices impact the human microbiome.

In this study, we analyzed the microbial diversity and metabolomic profiles of the saliva from 161 healthy individuals who had been following an omnivore, an ovo-lacto-vegetarian or a vegan diet for at least one year. We investigated whether dietary habits can have an impact in shaping the salivary microbiota and metabolome and whether they can alter its composition, creating a potential predisposition to disease.

## Results

In this study, we analyzed the microbial diversity and metabolomic profiles of the saliva from 161 healthy individuals who followed an omnivore, an ovo-lacto-vegetarian or a vegan diet. We sequenced amplicons of the V1–V3 regions of the 16S rRNA gene. Moreover, we analysed the salivary metabolome through ^1^H-NMR and GC-MS/SPME.

### Microbial diversity and a core microbiome in saliva

A total of 1,218,865 raw V1–V3 16S rRNA gene sequences were obtained through 454 pyrosequencing; 904,233 reads passed the filters of QIIME split_library.py script, with an average value of 5,616 reads/sample and an average length of 496 bp. The results of the α-diversity analysis are reported in Table S1. The Good’s Estimated Sample Coverage was above 97% in 90% of the samples (range 92–98%). A high level of microbial diversity was found in the saliva samples, with an average number of estimated Operational Taxonomic Units (OTUs) of 313±87.3. A Kruskal-Wallis non-parametric ANOVA did not show significant differences (P>0.05) between alpha-diversity parameters in the saliva of omnivore, ovo-lacto-vegetarian or vegan individuals.

**Figure 1:**
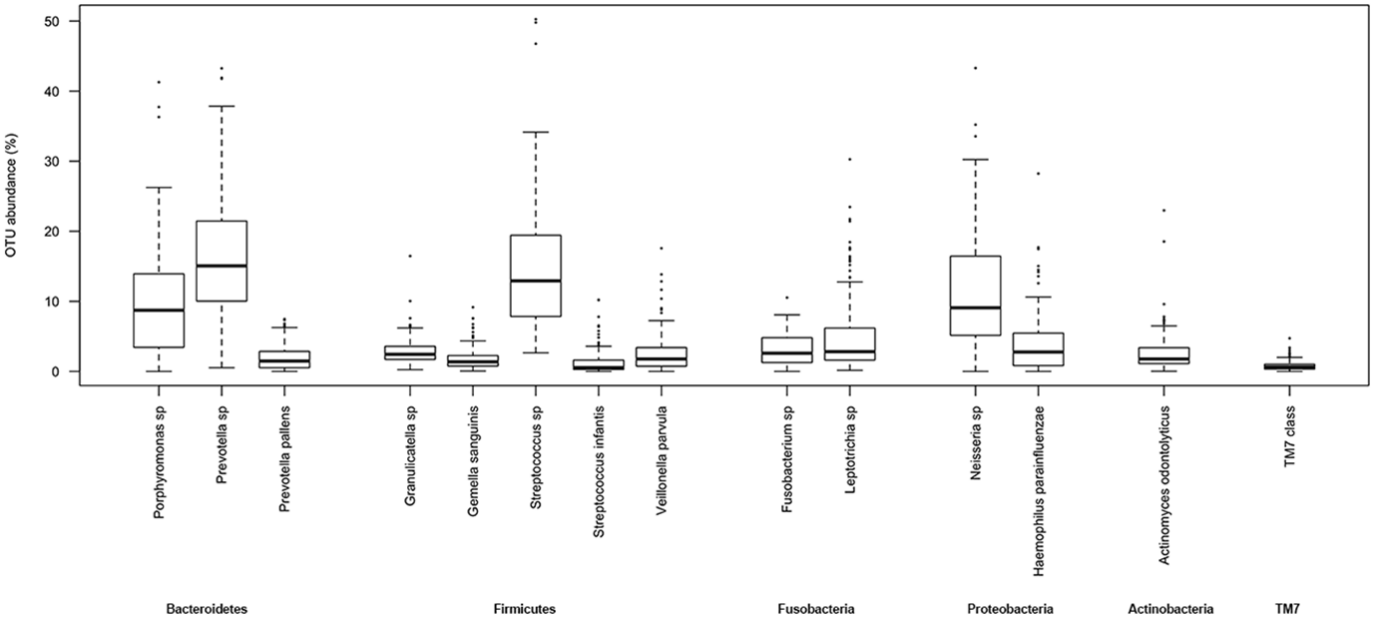
Abundance (%) of the 14 bacterial species identified in 98% of the saliva samples (n = 161). Boxes represent the interquartile range (IQR) between the first and third quartiles, and the line inside represents the median (2nd quartile). Whiskers denote the lowest and the highest values within 1.5×IQR from the first and third quartiles, respectively. Circles represent outliers beyond the whiskers. The boxes are grouped according to phylum.

OTUs were picked at 99% of similarity and two different databases were used for the taxonomic assignment. Since the results were not significantly different as measured by Wilcoxon-Mann-Whitney tests (P>0.05), the OTU table obtained through the Greengenes taxa assignment was used for the subsequent analyses. A core salivary microbiota was defined at the species level, including 14 OTUs, and occurred in at least 98% of the individuals (Table S2). The genera belonged to the phyla Bacteroidetes, Firmicutes, Fusobacteria, Proteobacteria, Actinobacteria and TM7 (Figure 1). Although the genera reported in Figure 1 were common to almost all the saliva samples, their abundances varied according to the individual. The box plot shows a considerable inter-individual variability of abundance. Only *Prevotella* sp. and *Streptococcus* sp. had a median relative abundance >10% (Figure 1). The core group occurring in 100% of the individuals included *Actinomyces odontolyticus, Prevotella* sp., *Granulicatella* sp., *Gemella sanguinis, Streptococcus* sp. and *Leptotrichia* sp. (Table S2).

### The salivary microbiota is not correlated to dietary habit

Using ADONIS, ANOSIM and MRPP methods, we observed no effect of dietary habit on the salivary microbiota (P<0.001). Accordingly, the β–diversity through weighted and unweighted UniFrac distance matrices did not show a separation between the omnivore, ovo-lacto-vegetarian or vegan individuals (Figure S1). Similarly, no influence of sex, age, site of collection or BMI was observed (P>0.05).

**Figure 2:**
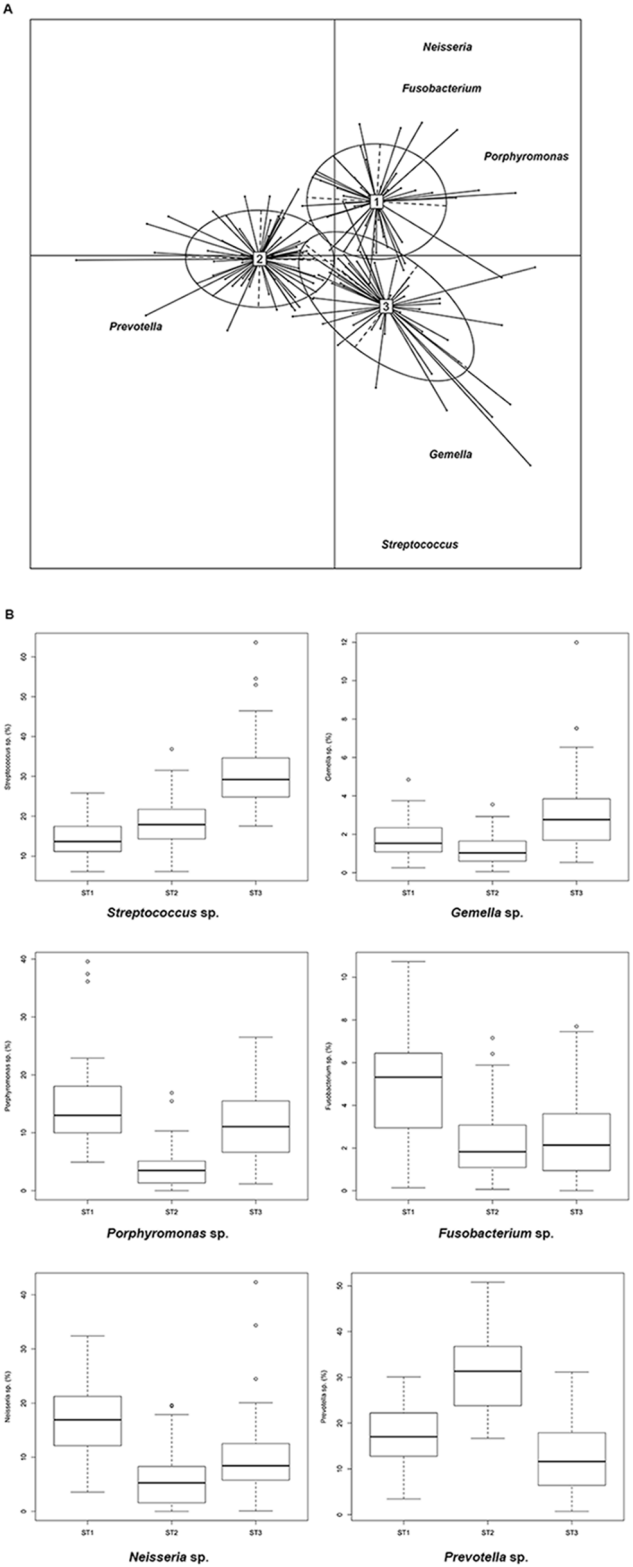
OTU abundance drives the differentiation of salivary types. (**A**) Between-class analysis visualizing the results from PCA and clustering based on the Jensen-Shannon distance of the saliva samples analyzed in this study (n = 161) showing a stratification of the samples into three salivary types (ST). The two principal components were plotted using the ade4 package in R with each sample represented by a circle. The center of gravity for each cluster is marked by a rectangle indicating the ST, and the ellipse covers 67% of the samples belonging to the cluster. Only those OTUs which showed a loading score > = 0.7 are shown in the figure. (**B**) Abundance (%) of the main contributors of each salivary type. See Fig. 1 for a definition of the box plot.

A PAM cluster analysis of the saliva samples was performed, and the Calinski-Harabasz index indicated that the optimal number of clusters was three. The cluster analysis indicated that the abundance of the core genera could drive the grouping of the individuals in three “salivary types”: cluster I was characterized by a higher abundance of *Neisseria* and *Fusobacterium*; cluster II was distinguished by *Prevotella* and cluster III by *Streptococcus* and *Gemella*; *Porphyromonas* was associated with both clusters I and III (Figure 2). Each of the above genera dominated in the corresponding cluster. As shown in the box plots, the median abundance of *Prevotella* and *Streptococcus* was approximately 30% in clusters 2 and 3, respectively (Figure 2). The taxa composition of the three “salivary types” were found to be significantly different using ADONIS (P<0.001) and ANOSIM (P<0.001). No significant association was found between dietary habit and “salivary type” (P>0.05), and each of the PAM clusters included saliva samples of the omnivore as well as the ovo-lacto-vegetarian and vegan individuals.

### OTU co-occurrence and/or co-exclusion

**Figure 3:**
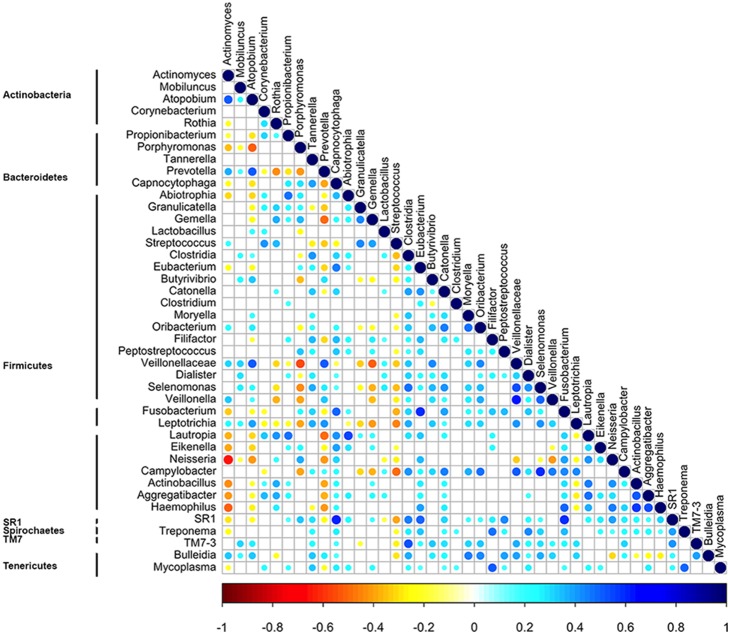
Significant co-occurrence and co-exclusion relationships between bacterial OTUs. Spearman’s rank correlation matrix of OTUs with > = 0.1% abundance in at least 5 saliva samples. Strong correlations are indicated by large circles, whereas weak correlations are indicated by small circles. The colors of the scale bar denote the nature of the correlation, with 1 indicating a perfectly positive correlation (dark blue) and −1 indicating a perfectly negative correlation (dark red) between two microbial genera. Only significant correlations (FDR<0.05) are shown.

The OTU co-occurrence was investigated by considering the genus-level taxonomic assignment and significant correlations at FDR<0.05 (Figure 3). *Streptococcus* showed the highest number of negative correlations, including the core-OTUs *Fusobacterium* and *Leptotrichia*. Within the core-OTUs, *Granulicatella* and *Gemella* co-occurred; they were correlated negatively to *Prevotella* and positively to *Streptococcus*. *Actinomyces* showed multiple negative correlations, including to *Neisseria* and *Haemophilus*. *Porphyromonas* was co-excluded with *Prevotella* and *Veillonellaceae*. The members of SR1 showed a co-occurrence with *Capnocytophaga* and TM7 with *Clostridia*. Taken together, the genera of *Fusobacteriaceae*, *Lachnospiraceae* and *Campylobacteriaceae* showed positive associations (Figure 3). Overall, the most abundant core genera that determined the stratification of the “salivary types” (Figure 2) showed a consistent mutual co-exclusion.

### Metabolome profiles

Metabolic profiling through ^1^H-NMR and GC-MS/SPME analyses detected and identified 49 (organic acids, free amino acids, monosaccharides and short-chain fatty acids) and 81 (alcohols, phenols, aldehydes, esters, ketones, hydrocarbons, aromatic heterocyclic compounds, sulfur compounds, tiophenes and terpenes) compounds, respectively (data not shown). No significant differences were found in the metabolite concentrations according to age, sex, site of collection or BMI (P>0.05).

**Figure 4:**
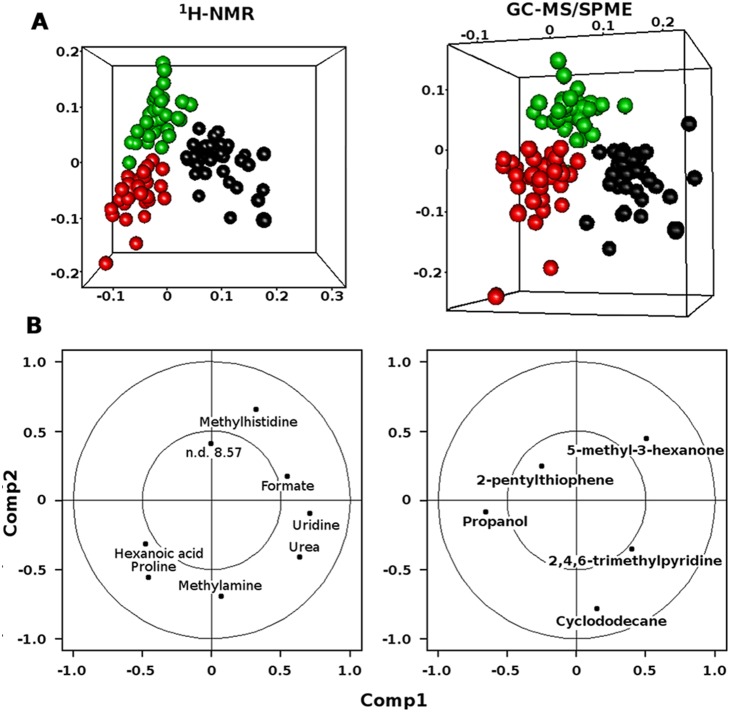
PLS-DA models based on ^1^H-NMR and GC-MS/SPME data. Score plots (**A**) and the corresponding loading plots (**B**) of PLS-DA models based on ^1^H-NMR and GC-MS/SPME data for the saliva from omnivore (black), ovo-lacto-vegetarian (green) and vegan (red) individuals. The circles identify the molecules with loading values between 0.5 and 1.

PLS-DA regression based on the ^1^H-NMR and Gas-chromatography mass spectrometry-solid-phase microextraction (GC-MS/SPME) significantly different compounds (P<0.05) resulted in models allowing a significant discrimination of the 3 groups of individuals with 2 latent variables (Figure 4). The correct classification rates for the omnivore, vegan and ovo-lacto-vegetarian individuals were 0.69, 0.67 and 0.64 for the ^1^H-NMR profiles and 0.64, 0.60 and 0.62 for the GC-MS/SPME profiles, respectively. Discrimination based on the ^1^H-NMR data was ascribed mainly to formate, urea and uridine, which showed higher levels in the omnivore individuals, and to hexanoic acid and proline, with higher levels in the non-omnivores. Moreover, the differentiation within the two non-omnivore groups was mainly attributed to methyl-amine, which was more abundant in the saliva of the vegan subjects, and methyl-histidine, which showed the highest levels in the ovo-lacto-vegetarians (Figure 4). The compounds from the GC-MS/SPME spectra with the highest discriminative ability included 1-propanol for the non-omnivores and 5-methyl-3-hexanone for the omnivores (Figure 4).

### OTU-metabolite co-occurrence and co-exclusion

**Figure 5:**
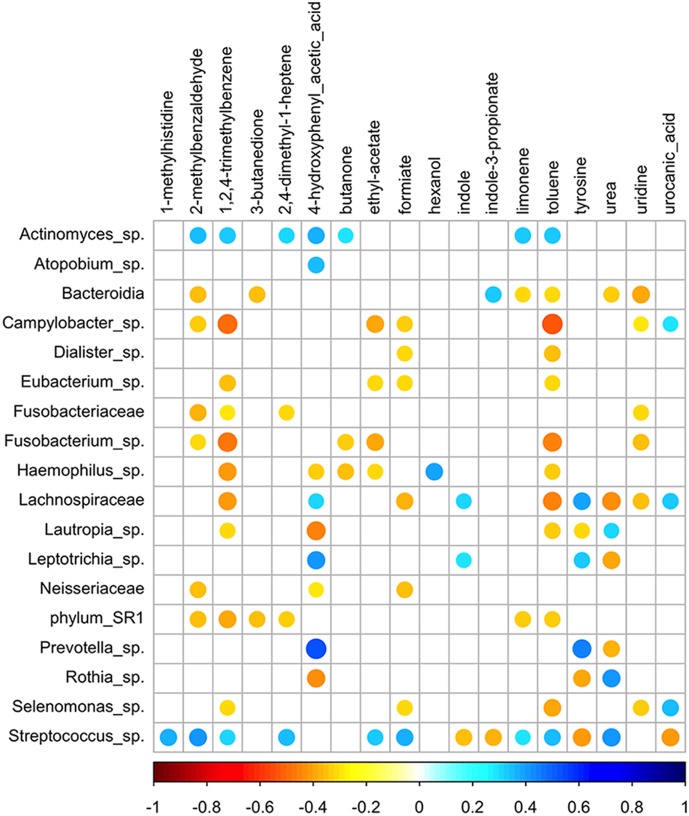
Significant co-occurrence and co-exclusion relationships between bacterial OTUs and metabolites. Spearman’s rank correlation matrix of significant relationships between OTUs (at genus level) and metabolites. Strong correlations are indicated by large circles, whereas weak correlations are indicated by small circles. The colors of the scale bar denote the nature of the correlation, with 1 indicating a perfectly positive correlation (dark blue) and −1 indicating a perfectly negative correlation (dark red) between microbial genera and metabolites. Only significant correlations (FDR<0.05) are shown.

All the significant (FDR<0.05) correlations between salivary OTUs and metabolites are reported in Figure 5. *Streptococcus* had the highest number of significant correlations, with soluble and volatile metabolites detected in the saliva samples. In particular, *Streptococcus* was positively correlated with 1-methyl-histidine, methyl-benzaldehyde and urea (Figure 5). The occurrence of *Prevotella* was significantly associated with 4-hydroxyphenylacetic acid (4HPA) and tyrosine. *Actinomyces* showed positive, although weak, correlations with seven different molecules, whereas many different OTUs were negatively correlated with 1,2,4-trimethylbenzene and toluene (Figure 5).

## Discussion

### Negligible effect of dietary habits on the salivary microbiome

We demonstrated that long-term dietary habits have no effect in shaping the salivary microbiota. To the best of our knowledge, data on the salivary microbiome as affected by diet had not been available. Recent advances in human microbiome studies have shown how diet can promptly cause changes in the gut microbiome [27], though such evidence was gathered by inducing a forced short-term diet change in human subjects and was not a comparison between different long-term dietary habits. The individuals recruited in the present study followed the same type of diet for at least one year; they could be regarded as habitual omnivores, ovo-lacto-vegetarians or vegans, and thus be studied to infer the consequences of prolonged dietary lifestyle.

In agreement with other studies [17], [18], [28], [29], the alpha diversity analysis showed that the saliva samples were complex ecosystems characterized by a high number of OTUs. A core microbiota, as defined by the Human Microbiome consortium [30], was identified in the present study. Other studies showed the presence of a core microbiota in the saliva of healthy individuals [17], [18], [28], and compared to other body sites, the oral environment appears to have the largest core [29]. Stahringer et al. [17] found a core of 8 genera in >95% of the saliva samples from 107 healthy individuals. A larger core, consisting of 12 different genera in >98% of individuals, was found in our study. In addition to the 8 genera shown previously [17], *Porphyromonas*, *Leptotrichia*, *Haemophilus, Actinomyces* and the TM7-3 class were also found as members of the core *taxa*. The classical periodontal pathogen *Aggregatibacter actinomycetemcomitans* [31], [32], [33] was identified only in one subject. *Treponema socranskii* and *Porphyromonas gingivalis*, reported in patients with periodontal diseases [32], [34], were found in 31 and 3 individuals out of 161, respectively, although they were never abundant (<0.1%). Overall, in none of the subjects the salivary microbiota suggested a state of oral disease and none of the disease-related OTUs was significantly associated to a specific dietary habit (P>0.05).

It should be pointed out that these results can be influenced by the OTU clustering methodology. Recent studies showed that oligotyping (a new clustering-free approach) was able to detect a higher number of species than traditional OTU clustering techniques and that each OTU can be split into several distinct subpopulations, all ecologically distinct but with 16S tags differing by as little as one nucleotide [35], [36].

To the best of our knowledge, no studies exist about the influence of dietary choices on salivary microbiota and about the possible effect of a long-term vegetarian or vegan diets in inducing changes in the microbial composition/abundance, compared to a “typical” omnivore oral microbiota. Overall, our results excluded that vegan or ovo-lacto-vegetarian diets could significantly impact on the salivary microbiota.

### Abundances of core microbial taxa lead to “salivary types” and co-occurrence patterns

The abundance of some of the core genera across the samples resulted in a grouping of the individuals by PAM clustering. Although the analysis showed a high similarity within the population, and the three clusters shared a certain number of microbial genera, three possible “salivary types” were distinguished including: the core microbial genera [I] *Neisseria*-*Fusobacterium*, [II] *Prevotella* and [III] *Streptococcus-Gemella*. Therefore, although the core genera were common to the saliva of all the subjects, the abundance of Fusobacteria/Proteobacteria, Bacteroidetes and Firmicutes enabled a differentiation between the individuals. The three clusters included individuals independently on diet, age, sex, site of collection or BMI. The same clustering method has been used to identify enterotypes from the human gut microbiota [37]. Nevertheless, very recently, it was noted that the groupings obtained should not be considered as discrete clusters but as a simplified stratification of the samples [38]. In our study, PAM analysis indicated that the individuals had a trend of differentiation based on the relative abundance of *taxa* belonging to the core microbiota. Similarly, although not identically, a clustering of saliva samples was found through the analysis of 16S rRNA genes by non-sequencing-based approaches [39]: the clusters were linked to oral health, and *Prevotella* and *Streptococcus* clusters were associated with wider periodontal pockets.

The microbial co-occurrence/exclusion pattern is suggested to be specific for each body site: it is particularly evident for the oral cavity and is correlated to phylogenetic relatedness [40]. Species with similar nutritional needs tend to co-occur according to the availability of the specific nutrients [41]. The results of our study indicate that the core microbial genera of Firmicutes (including *Streptococcus*, *Gemella* and *Granulicatella*) or Bacteroidetes (*Prevotella* and *Porphyromonas*) tend to dominate the salivary microbiota and to exclude other bacterial *taxa*. Furthermore, co-exclusion was found between Proteobacteria (*Neisseria* and Pasteurellaceae) and Actinobacteria (*Actinomyces*). The cause and consequences of such co-occurrence/exclusions certainly include nutrient consumption, metabolite release and synergism/antagonism dynamics [40]. Details on such mechanisms are still unresolved. However, the relationships between microbial populations, as highlighted in the healthy individuals of this study, suggest that equilibrium among species exists regardless of dietary lifestyle.

### Salivary metabolome differentiates dietary habits

Most of the detected compounds identified in this study are consistent with those previously reported in the saliva of healthy individuals [23], [42]–[45]. Their possible entry routes into the salivary flow are very diverse and can include environmental exposure through the inhalation of air and/or water vapor ingestion, food intake, transdermal absorption, blood, oro-nasal tissues and gastrointestinal reflux.

Using ^1^H-NMR- and GC-MS/SPME-based metabolomics, this study revealed differences in the salivary metabolites of healthy individuals, which correlated with dietary habits. Indeed, several compounds detected through both techniques were found to be significantly different among the three diet groups. PLS-DA models built on their concentration were able to discriminate between omnivore and non-omnivore individuals, so that dietary habits of the individuals could be correctly predicted in 70% of the cases. This differentiation is ascribed to the different levels of various molecules, such as urea, uridine and formate. Although the origin of some compounds is unclear, others may be attributed to human physiological metabolism. A contribution of oral microbiota metabolism and food intake can also be hypothesized, although it is clear that the salivary taxonomic composition is not linked to diet and presumably does not contribute to the metabolomic differentiation of the saliva samples. Urea is derived from protein digestion, and it has been widely recognized as a biomarker of animal protein consumption in addition to creatine, creatinine, histidine and trimethylamine-N-oxide [46], [47]. Urea is released continuously in salivary secretions, and its hydrolysis by oral bacteria is the primary route for generating alkali in dental plaque, which is important for oral homeostasis and caries prevention [48]. Indeed, urea is the main factor responsible for the basic pH of the saliva of omnivores [49]. The acidic saliva of non-omnivores, with particular reference to vegans, has been indicated as the main cause of the high incidence of dental lesions, together with the mechanical action of chewing highly fibrous food [50].

Significantly higher levels of formate were observed in omnivores. This may be related to the activity of the oral bacteria that can degrade nitrogenous compounds associated with a meat-rich diet into small peptides and amino acids for subsequent use. The downstream amino acid degradation can generate formate. However, the OTU-metabolite correlation analysis revealed a very weak correlation between formate and microbial species. Among the volatile compounds, only 1-propanol discriminated the saliva of non-omnivore individuals. It is considered to be the result of the bacterial fermentation of threonine and isoleucine, essential amino acids present at high levels in several legumes and vegetables [51], [52]. However, because alcohols are primary volatiles from vegetables, 1-propanol may be directly derived from the intake of vegetable foods.

### The presence of some molecules correlates with salivary microbiota

The correlation between OTU occurrence and metabolites was evaluated in this study. However, only few of the correlations found included molecules that were discriminative of the diets based on PLS-DA. This was not surprising, given the lack of correlation between microbiota and dietary habit. Nevertheless, a map of significant correlations between bacterial OTUs and metabolites was drawn (Figure 5). 4HPA is a metabolite of the catabolism of free aromatic amino acids. *Prevotella* was positively associated with the occurrence of 4HPA and, consistently, with tyrosine. 4HPA in saliva is regarded as a signal molecule involved in the regulation of adhesin expression in *Neisseria meningitidis* [53]. The correlations between 4HPA and nine different genera may indicate a possible role of this molecule in the regulatory systems of other bacteria in addition to *Neisseria*. Finally, 1,2,4-trimethylbenzene and toluene, defined as contaminants of concern [54], [55], showed an almost identical pattern of correlation with several OTUs. Such a negative correlation suggests that there can be an inhibitory effect of aromatic hydrocarbons on the oral microbiota.

## Conclusions

Saliva provides useful diet-related biomarkers that can discriminate omnivore and non-omnivore dietary habits.

The structure of the salivary microbiota is not influenced by dietary habits and is instead characterized by a richness in bacterial *taxa* and inter-individual similarity, with a large core of bacterial genera. Based on the available literature, the “healthy” status of individuals appears to have a strong influence on the structure of oral microbial populations. Microbial homeostasis could be perturbed in the case of sub-standard oral hygiene or other unascertained environmental factors, but at present there is no indication that a choice of omnivore, ovo-lacto-vegetarian or vegan diet can lead to specific traits in the oral microbiota.

## Materials and Methods

A detailed description of the methods is provided as supporting information (Methods S1).

### Recruitment and sample collection

Adult healthy volunteers (n = 161) aged 18–55 (38±9.8), with BMI>18 (22±2.3), following a habitual omnivore (total n = 55), ovo-lacto-vegetarian (total n = 55) or vegan (total n = 51) diet were recruited at 4 different sites in Italy. The collection centers were in Bari, Bologna, Parma and Torino. The subjects had been following an omnivore, ovo-lacto-vegetarian or vegan diet for at least one year at the time of recruitment. Males constituted 35%, 45% and 45% of the recruited omnivore, ovo-lacto-vegetarian and vegan volunteers, respectively.

Unstimulated whole saliva was collected into sterile Falcon tubes (50 mL), as recently described [10]. After the collection of 5 mL of resting saliva (maximum collection time fixed at 30 min) the samples were stored at −20°C. Saliva samples were collected on three different days of three consecutive weeks, and the three samples were pooled before the microbiota and metabolome analyses.

### Microbial diversity analysis

Microbial DNA extraction was carried out using the Biostic Bacteremia DNA isolation kit (MoBIO Laboratories, Inc. Carlsbad, CA) with 2 mL of saliva sample. The microbial diversity was assessed by pyrosequencing of the amplified (520 bp) V1–V3 region of the 16S rRNA gene using a 454 GS Junior platform (454 Life Sciences, Roche Diagnostics, Italy). Library preparation and sequencing were carried out as previously described [56].

Raw reads were first filtered according to the 454 processing pipeline. The sequences were then analyzed and further filtered using QIIME 1.8.0 software [57]. A quality filter of the sequences was applied using the split library script by QIIME, as recently described [58]. OTUs were picked at 99% of similarity and representative sequences of each cluster were used to assign taxonomy. Two different databases were used: the Greengenes [59] and the QIIME formatted version of the Human Oral Microbiome Database (HOMD) [60]. Alpha and beta diversity and statistical analyses were carried out in QIIME, as reported elsewhere [58].

Correlation analysis, sample clustering and statistical analyses were carried out in R environment (www.r-project.org).

### ^1^H nuclear magnetic resonance (NMR) spectroscopy and gas-chromatography mass spectrometry-solid-phase microextraction (GC-MS/SPME) analyses

Samples for NMR analysis were prepared according to Mikkonen et al. [61]. All ^1^H-NMR spectra were recorded at 300 K using a Bruker US+ Avance III spectrometer operating at 600 MHz (Bruker BioSpin, Karlsruhe, Germany). For this purpose, the first increment of the nuclear overhauser effect spectroscopy pulse sequence [62] was employed, with a relaxation delay of 7 sec and an acquisition time of 2.28 sec. The residual HDO signal was reduced through presaturation. The spectra were corrected for errors in chemical shift misalignments using an interval correlation optimized shifting procedure [63]. Signal assignment was carried out on the basis of the literature [23] and using the Amix software (version 2.1.3, Bruker BioSpin). The spectra were finally averaged over portions of 0.018 ppm.

GC-MS/SPME analysis was performed as previously described [64] and modified as follows. The samples were equilibrated for 10 min at 50°C with stirring. The temperature program was as follows: 40°C for 1 min and then programmed at 4.5°C rise/min to 65°C and finally at 10°C rise/min to 230°C, which was maintained for 17 min. All the GC-MS raw files were converted to netCDF format via Chemstation (Agilent Technologies) and subsequently processed with the XCMS toolbox (http://metlin.scripps.edu/download/). The GC-MS/SPME data were exported into R for subsequent statistical or multivariate analyses.

### Statistical analysis of GC-MS/SPME and ^1^H-NMR data

To account for possible different natural dilutions of the samples, both the ^1^H-NMR spectra and GC-MS/SPME data were normalized using probabilistic quotient normalization [65].

Molecules with different concentrations in relation to the three diets were then searched by means of an analysis of variance (ANOVA), followed by an LSD test (P<0.05). The necessary calculations were performed using R software.

To discriminate the ^1^H-NMR or GC-MS/SPME profiles as a function of diet, models based on projection on latent structures (PLS) in its discriminant (DA) version [66] were built based on the normalized concentration of the significant molecules identified. Twenty-five percent of the samples pertaining to each dietary group was randomly chosen to be employed as a training set to test the robustness of the model. The optimal number of latent variables was calculated on the training set using the leave-10-out method. The random division of the samples into training and test sets and the consequent creation of the predictive model were repeated 100 times, and the parameters of robustness were averaged. The calculations were performed using the R package mixOmics (www.r-project.org). Moreover, ANOVA (for discrete variables) or Spearman’s rank correlation test (for continuous variables) were carried out in order to define a possible effect of sex, site of collection, age and BMI on the metabolite concentration.

### Nucleotide sequence accession number

The 16S rRNA gene sequences are available at the Sequence Read Archive of NCBI (accession number SRP035877).

### Ethics statement

All participants were informed about the study aims and provided informed written consent. The study was approved by the Ethics Committee of (i) Azienda Sanitaria Locale (Bari) (protocol N.1050), (ii) Azienda Ospedaliera Universitaria of Bologna (protocol N.0018396), (iii) Province of Parma (protocol N.22884) and (iv) University of Torino (protocol N.1/2013/C).

## Supporting Information

Figure S1 **Principal Coordinates Analysis of jackknifed weighted (A) and unweighted (B) UniFrac distances for 16S rRNA gene sequence data.** Principal coordinate plot of the first three components showing no differences between omnivore (red dots), ovo-lacto-vegetarian (orange dots) and vegan (blue dots) subjects.
(TIF)

Table S1 Observed diversity and estimated sample coverage for 16S rRNA amplicons analyzed in this study.
(DOCX)

Table S2 Average abundance, minimum and maximum value and prevalence (%) of the core OTUs in the 161 saliva samples analysed.
(DOCX)

Methods S1 **Supporting material and methods.**
(DOCX)
