# Supplementary figures and images for: The Same Microbiota and a Potentially Discriminant Metabolome in the Saliva of Omnivore, Ovo-Lacto-Vegetarian and Vegan Individuals
Source: PLoS One. 2014 Nov 5;9(11):e112373. doi: 10.1371/journal.pone.0112373 (PMC4221475; doi:10.1371/journal.pone.0112373)

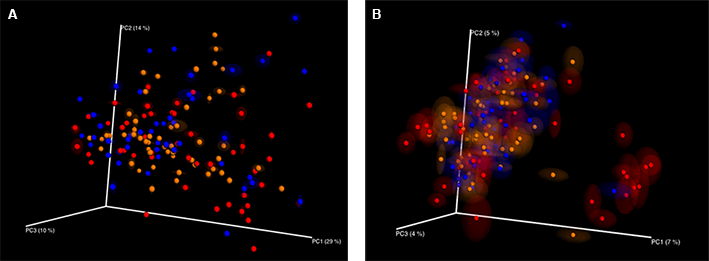

Supplement: Figure S1 — Principal Coordinates Analysis of jackknifed weighted (A) and unweighted (B) UniFrac distances for 16S rRNA gene sequence data. Principal coordinate plot of the first three components showing no differences between omnivore (red dots), ovo-lacto-vegetarian (orange dots) and vegan (blue dots) subjects. (TIF) [file pone.0112373.s001.tif]
