# Supplementary material for: The Same Microbiota and a Potentially Discriminant Metabolome in the Saliva of Omnivore, Ovo-Lacto-Vegetarian and Vegan Individuals
Source: PLoS One. 2014 Nov 5;9(11):e112373. doi: 10.1371/journal.pone.0112373 (PMC4221475; doi:10.1371/journal.pone.0112373)
Supplement: Table S1 — Observed diversity and estimated sample coverage for 16S rRNA amplicons analyzed in this study. (DOCX) [file pone.0112373.s002.docx]

**Table S1.** Observed diversity and estimated sample coverage for 16S rRNA amplicons analyzed in this study.

| **Sample ID** | **OTUs** | **Chao1** | **Shannon** | **ESC** |
| --- | --- | --- | --- | --- |
| 09-BA-O | 237 | 487.10 | 4.88 | 97.14 |
| 13-BA-O | 250 | 399.88 | 5.01 | 97.71 |
| 04-BA-O | 94 | 154.06 | 3.75 | 96.08 |
| 10-BA-O | 245 | 423.36 | 4.55 | 97.92 |
| 14-BA-O | 432 | 694.97 | 5.23 | 98.62 |
| 01-BA-O | 114 | 175.89 | 5.30 | 95.11 |
| 02-BA-O | 290 | 515.08 | 5.33 | 97.25 |
| 11-BA-O | 106 | 165.13 | 5.28 | 94.78 |
| 08-BA-O | 170 | 325.88 | 4.81 | 95.59 |
| 03-BA-O | 205 | 322.00 | 4.98 | 96.65 |
| 15-BA-O | 285 | 472.50 | 5.70 | 97.25 |
| 07-BA-O | 300 | 491.57 | 4.78 | 96.98 |
| 12-BA-O | 112 | 211.00 | 5.20 | 92.62 |
| 05-BA-O | 157 | 338.24 | 4.47 | 96.40 |
| 06-BA-O | 260 | 467.23 | 3.99 | 97.76 |
| 42-BO-O | 314 | 400.42 | 6.14 | 97.83 |
| 28-BO-O | 215 | 331.00 | 5.34 | 97.21 |
| 24-BO-O | 338 | 471.05 | 4.40 | 98.27 |
| 23-BO-O | 382 | 487.85 | 5.71 | 97.46 |
| 29-BO-O | 395 | 545.75 | 5.80 | 97.19 |
| 30-BO-O | 469 | 611.92 | 6.14 | 97.27 |
| 08-BO-O | 379 | 510.21 | 5.88 | 96.74 |
| 03-BO-O | 415 | 527.54 | 5.36 | 98.24 |
| 06-BO-O | 371 | 486.73 | 5.01 | 98.13 |
| 16-BO-O | 344 | 437.19 | 4.81 | 98.27 |
| 31-BO-O | 355 | 515.38 | 5.37 | 96.91 |
| 20-BO-O | 484 | 583.70 | 5.76 | 98.22 |
| 21-BO-O | 267 | 337.60 | 5.60 | 96.77 |
| 22-BO-O | 336 | 431.88 | 5.29 | 97.26 |
| 09-BO-O | 389 | 463.51 | 5.13 | 98.69 |
| 36-PR-O | 312 | 375.53 | 5.73 | 98.67 |
| 31-PR-O | 329 | 426.28 | 6.14 | 97.88 |
| 34-PR-O | 286 | 396.00 | 5.28 | 98.19 |
| 01-PR-O | 248 | 337.76 | 5.31 | 98.28 |
| 37-PR-O | 286 | 377.24 | 5.47 | 98.43 |
| 29-PR-O | 322 | 382.77 | 6.01 | 98.69 |
| 39-PR-O | 297 | 357.33 | 5.74 | 98.64 |
| 25-PR-O | 344 | 441.63 | 5.37 | 98.31 |
| 19-PR-O | 329 | 398.28 | 5.27 | 98.67 |
| 38-PR-O | 284 | 350.98 | 5.54 | 98.62 |
| 22-TO-O | 347 | 495.52 | 5.61 | 97.21 |
| 09-TO-O | 278 | 350.86 | 5.30 | 98.15 |
| 20-TO-O | 291 | 394.21 | 5.72 | 97.83 |
| 37-TO-O | 397 | 578.25 | 5.79 | 97.22 |
| 12-TO-O | 355 | 472.54 | 5.86 | 97.68 |
| 23-TO-O | 400 | 547.00 | 6.03 | 97.24 |
| 17-TO-O | 353 | 484.00 | 5.03 | 97.79 |
| 43-TO-O | 433 | 564.69 | 4.75 | 98.53 |
| 14-TO-O | 317 | 452.02 | 5.54 | 97.72 |
| 15-TO-O | 456 | 607.01 | 5.30 | 98.35 |
| 31-TO-O | 288 | 393.02 | 4.40 | 98.48 |
| 18-TO-O | 439 | 613.25 | 5.65 | 97.46 |
| 33-TO-O | 355 | 538.18 | 5.45 | 97.07 |
| 16-TO-O | 378 | 519.44 | 5.17 | 98.07 |
| 13-TO-O | 469 | 571.75 | 6.22 | 98.34 |
| 07-BA-V | 194 | 240.58 | 5.28 | 98.61 |
| 06-BA-V | 304 | 419.11 | 5.07 | 98.18 |
| 05-BA-V | 296 | 397.06 | 5.22 | 98.33 |
| 04-BA-V | 53 | 76.00 | 3.68 | 95.30 |
| 01-BA-V | 142 | 355.00 | 5.04 | 95.59 |
| 08-BA-V | 292 | 427.47 | 4.88 | 97.25 |
| 10-BA-V | 116 | 169.13 | 4.15 | 96.75 |
| 02-BA-V | 92 | 174.50 | 4.44 | 95.11 |
| 13-BA-V | 116 | 204.50 | 4.86 | 92.41 |
| 09-BA-V | 394 | 519.78 | 5.14 | 97.46 |
| 03-BA-V | 242 | 343.06 | 5.96 | 94.47 |
| 12-BA-V | 310 | 425.00 | 5.20 | 97.01 |
| 11-BA-V | 304 | 397.19 | 4.46 | 97.86 |
| 44-BO-V | 299 | 377.53 | 5.58 | 98.57 |
| 45-BO-V | 256 | 335.33 | 5.03 | 98.36 |
| 36-BO-V | 300 | 372.02 | 5.08 | 98.31 |
| 26-BO-V | 286 | 420.37 | 4.88 | 98.09 |
| 07-BO-V | 344 | 434.55 | 5.54 | 98.27 |
| 02-BO-V | 307 | 405.10 | 5.25 | 98.02 |
| 18-BO-V | 384 | 527.01 | 5.39 | 98.09 |
| 12-BO-V | 367 | 506.14 | 4.94 | 97.64 |
| 11-BO-V | 366 | 478.32 | 4.81 | 98.60 |
| 10-BO-V | 297 | 408.10 | 5.33 | 97.21 |
| 35-BO-V | 360 | 458.61 | 5.63 | 97.53 |
| 13-BO-V | 346 | 434.58 | 4.91 | 97.95 |
| 33-PR-V | 186 | 270.00 | 5.39 | 97.55 |
| 32-PR-V | 289 | 371.50 | 5.76 | 97.83 |
| 35-PR-V | 355 | 426.30 | 6.08 | 98.33 |
| 22-PR-V | 343 | 493.11 | 5.60 | 97.66 |
| 21-PR-V | 177 | 240.41 | 4.62 | 97.25 |
| 26-PR-V | 309 | 442.17 | 5.45 | 98.26 |
| 23-PR-V | 313 | 416.02 | 5.35 | 98.11 |
| 18-PR-V | 357 | 431.45 | 5.86 | 98.67 |
| 17-PR-V | 381 | 465.64 | 5.93 | 98.54 |
| 11-PR-V | 267 | 374.33 | 5.62 | 97.46 |
| 09-PR-V | 367 | 486.11 | 5.27 | 98.37 |
| 12-PR-V | 319 | 375.23 | 5.72 | 98.68 |
| 07-PR-V | 373 | 446.50 | 5.77 | 98.59 |
| 32-TO-V | 219 | 294.00 | 5.07 | 97.87 |
| 11-TO-V | 270 | 336.45 | 5.05 | 97.78 |
| 21-TO-V | 322 | 423.61 | 5.59 | 97.53 |
| 35-TO-V | 285 | 382.19 | 5.59 | 97.61 |
| 24-TO-V | 289 | 365.39 | 5.28 | 97.99 |
| 27-TO-V | 324 | 413.76 | 5.58 | 97.84 |
| 30-TO-V | 206 | 264.41 | 4.82 | 98.56 |
| 44-TO-V | 430 | 525.62 | 5.53 | 98.10 |
| 10-TO-V | 402 | 485.80 | 5.62 | 98.30 |
| 19-TO-V | 352 | 452.34 | 5.47 | 97.49 |
| 03-TO-V | 435 | 530.63 | 5.47 | 98.42 |
| 25-TO-V | 354 | 451.23 | 5.35 | 98.39 |
| 07-TO-V | 411 | 574.77 | 5.53 | 98.08 |
| 11-BA-VG | 331 | 434.25 | 5.06 | 98.16 |
| 10-BA-VG | 408 | 528.61 | 5.75 | 97.33 |
| 12-BA-VG | 277 | 354.92 | 4.82 | 98.20 |
| 14-BA-VG | 373 | 469.04 | 5.42 | 97.94 |
| 01-BA-VG | 265 | 350.43 | 4.75 | 98.50 |
| 02-BA-VG | 249 | 328.23 | 5.72 | 97.44 |
| 08-BA-VG | 379 | 465.25 | 5.66 | 97.77 |
| 03-BA-VG | 190 | 276.06 | 5.05 | 95.62 |
| 05-BA-VG | 254 | 314.76 | 4.94 | 97.64 |
| 07-BA-VG | 284 | 421.57 | 4.97 | 97.46 |
| 09-BA-VG | 359 | 448.08 | 5.31 | 97.33 |
| 06-BA-VG | 271 | 372.06 | 5.00 | 97.87 |
| 04-BA-VG | 295 | 383.06 | 5.06 | 97.30 |
| 25-BO-VG | 291 | 376.71 | 5.59 | 98.29 |
| 34-BO-VG | 373 | 504.01 | 5.91 | 97.41 |
| 01-BO-VG | 277 | 368.00 | 5.41 | 98.26 |
| 39-BO-VG | 255 | 334.33 | 4.90 | 98.35 |
| 37-BO-VG | 257 | 350.00 | 5.21 | 97.81 |
| 04-BO-VG | 233 | 355.16 | 5.72 | 95.62 |
| 19-BO-VG | 267 | 368.22 | 5.57 | 97.67 |
| 40-BO-VG | 343 | 456.11 | 5.81 | 97.88 |
| 32-BO-VG | 363 | 444.57 | 5.68 | 97.04 |
| 15-BO-VG | 418 | 512.80 | 5.60 | 97.90 |
| 17-BO-VG | 304 | 388.37 | 5.89 | 96.17 |
| 05-BO-VG | 371 | 498.22 | 4.95 | 98.25 |
| 27-BO-VG | 336 | 439.60 | 5.71 | 97.12 |
| 14-BO-VG | 481 | 584.88 | 5.74 | 98.52 |
| 30-PR-VG | 307 | 384.29 | 5.46 | 98.35 |
| 05-PR-VG | 294 | 357.89 | 5.72 | 98.51 |
| 02-PR-VG | 260 | 371.63 | 5.78 | 97.02 |
| 03-PR-VG | 274 | 361.13 | 5.47 | 97.42 |
| 04-PR-VG | 254 | 335.03 | 5.44 | 98.35 |
| 27-PR-VG | 350 | 433.08 | 6.08 | 98.32 |
| 15-PR-VG | 277 | 350.64 | 4.99 | 98.88 |
| 13-PR-VG | 328 | 451.17 | 5.45 | 98.44 |
| 20-PR-VG | 284 | 347.20 | 5.51 | 98.88 |
| 14-PR-VG | 294 | 383.02 | 5.46 | 98.33 |
| 06-PR-VG | 196 | 252.80 | 5.38 | 96.93 |
| 16-PR-VG | 360 | 491.10 | 5.93 | 98.04 |
| 08-PR-VG | 334 | 413.73 | 5.16 | 98.56 |
| 10-PR-VG | 155 | 216.22 | 4.02 | 98.18 |
| 02-TO-VG | 301 | 468.50 | 5.43 | 95.22 |
| 29-TO-VG | 392 | 533.00 | 5.47 | 97.45 |
| 04-TO-VG | 619 | 703.50 | 6.49 | 98.97 |
| 41-TO-VG | 481 | 643.25 | 5.69 | 97.53 |
| 34-TO-VG | 341 | 604.89 | 5.53 | 95.84 |
| 26-TO-VG | 387 | 534.70 | 5.59 | 98.01 |
| 42-TO-VG | 389 | 582.80 | 5.69 | 97.81 |
| 39-TO-VG | 341 | 468.10 | 4.79 | 98.28 |
| 06-TO-VG | 346 | 435.29 | 5.07 | 98.39 |
| 01-TO-VG | 385 | 487.27 | 4.98 | 98.58 |
| 28-TO-VG | 421 | 562.01 | 5.36 | 98.05 |
| 38-TO-VG | 399 | 505.16 | 5.80 | 98.19 |
| 05-TO-VG | 396 | 508.84 | 5.16 | 98.12 |
| 08-TO-VG | 390 | 521.60 | 5.56 | 97.56 |
