# Supplementary material for: The Same Microbiota and a Potentially Discriminant Metabolome in the Saliva of Omnivore, Ovo-Lacto-Vegetarian and Vegan Individuals
Source: PLoS One. 2014 Nov 5;9(11):e112373. doi: 10.1371/journal.pone.0112373 (PMC4221475; doi:10.1371/journal.pone.0112373)
Supplement: Table S2 — Average abundance, minimum and maximum value and prevalence (%) of the core OTUs in the 161 saliva samples analysed. (DOCX) [file pone.0112373.s003.docx]

| **OTU** | **Mean** | **Min** | **Max** | **Prevalence** |
| --- | --- | --- | --- | --- |
| *Porphyromonas* sp*.* | 9.50 | 0 | 41.27 | 98.8 |
| *Prevotella* sp. | 16.44 | 0.53 | 43.24 | 100 |
| *P. pallens* | 1.90 | 0 | 7.48 | 98.1 |
| *Granulicatella* sp*.* | 2.85 | 0.26 | 16.46 | 100 |
| *Gemella sanguinis* | 1.81 | 0.06 | 9.16 | 100 |
| *Streptococcus* sp. | 14.72 | 2.66 | 50.25 | 100 |
| *Str. infantis* | 1.32 | 0 | 10.22 | 99.4 |
| *Veillonella parvula* | 2.63 | 0 | 17.57 | 98.1 |
| *Fusobacterium* sp. | 3.16 | 0 | 10.53 | 99.4 |
| *Leptotrichia* sp. | 4.98 | 0.16 | 30.27 | 100 |
| *Neisseria* sp. | 11.03 | 0 | 43.29 | 99.4 |
| *Haemophilus parainfluenzae* | 3.93 | 0 | 28.23 | 98.1 |
| *Actinomyces odontolyticus* | 2.55 | 0.02 | 22.98 | 100 |
| TM7 class | 0.84 | 0 | 4.73 | 99.4 |

**Table S2 –** Average abundance, minimum and maximum value and prevalence (%) of the core OTUs in the 161 saliva samples analysed.
