# Supplementary material for: The Same Microbiota and a Potentially Discriminant Metabolome in the Saliva of Omnivore, Ovo-Lacto-Vegetarian and Vegan Individuals
Source: PLoS One. 2014 Nov 5;9(11):e112373. doi: 10.1371/journal.pone.0112373 (PMC4221475; doi:10.1371/journal.pone.0112373)
Supplement: Methods S1 — Supporting material and methods. (DOCX) [file pone.0112373.s004.docx]

**Supplementary Matherials and Methods**

**Recruitment and sample collection**

Adult healthy volunteers (n=161) aged 18-55 (38 ± 9.8), with BMI>18 (22 ± 2.3), following a habitual omnivore (total n=55), ovo-lacto-vegetarian (total n=55) or vegan (total n=51) diet were recruited at 4 different sites in Italy. The collection centers were in Bari, Bologna, Parma and Torino. Males constituted 35%, 45% and 45% of the recruited omnivore, ovo-lacto-vegetarian and vegan volunteers, respectively. During a preparative consultation interview, the candidates were informed of the scope of the research; all the subjects gave written informed consent, and the study was approved by the Ethics Committee of (i) Azienda Sanitaria Locale (Bari) (protocol N.1050), (ii) Azienda Ospedaliera Universitaria of Bologna (protocol N.0018396), (iii) Province of Parma (protocol N.22884) and (iv) University of Torino (protocol N.1/2013/C).

Candidate volunteers were not enrolled in the following cases: if they had any diagnosed metabolic or systemic disease, were pregnant or nursing, or had been under probiotic or antibiotic (sometime in the last three months) treatment. When recruited, the volunteers were asked to record a daily food diary before and during sampling with the aim of double checking the current type of diet followed. The recruitment stopped if the dietary habit deviated from the self-proclaimed omnivore, ovo-lacto-vegetarian or vegan diet.

**Microbial diversity analysis**

Total DNA extraction from the saliva samples was carried out by using the Biostic^TM^ Bacteremia DNA isolation kit (MO BIO Laboratories, Inc. Carlsbad, CA). The protocol was applied to the pellet (12,000 g) of 2 ml of suspension. The microbial diversity was studied by pyrosequencing of the amplified V1-V3 region of the 16S rRNA gene by using primers Gray28F 5’-TTTGATCNTGGCTCAG and Gray519r 5’-GTNTTACNGCGGCKGCTG amplifying a fragment of 520 bp [1]. 454-adaptors were included in the forward primer followed by a 10 bp sample-specific Multiplex Identifier (MID). Each PCR mixture (final volume, 50 μl) contained 50 ng of template DNA, 0.4 μM of each primer, 0.50 mmol l^-1^ of each deoxynucleoside triphosphate, 2.5 mmol l^-1^ MgCl_2_, 5 μl of 10 X PCR buffer and 2.5 U of native *Taq* polymerase (Invitrogen, Milano, Italy). The following PCR conditions were used: 94°C for 2 min, 35 cycles of 95°C for 20 s, 56°C for 45 s and 72°C for 5 min, and a final extension at 72°C for 7 min. Each sample was amplified in duplicate, amplicons were pooled and purified twice by using the Agencourt AMPure kit (Beckman Coulter, Milano, Italy), quantified by using a QuantiFluor^TM^ (Promega, Milano, Italy) and an equimolar pool was obtained prior to further processing. The amplicons were used as a template for pyrosequencing on a GS Junior platform (454 Life Sciences, Roche Diagnostics, Italy) according to the manufacturer’s instructions by using a Titanium chemistry. Moreover, 15 samples were sequenced in replicate and since no significant difference was found between the technical replicates, we did not include these results.

**Bioinformatics and sequencing data analysis**

In order to guarantee a higher level of accuracy in terms of Operational Taxonomic Units (OTUs) detection, after the split library script performed by QIIME, the reads were excluded from the analysis if they had an average quality score lower than 25, if they were shorter than 300 bp and if there were ambiguous base calls. After filtering and denoising [2], OTUs defined by a 99% of similarity were picked using the uclust method [3] and the representative sequences were submitted to the RDPII classifier [4] to obtain the taxonomy assignment using both the Greengenes 16S rRNA gene database [5] and the QIIME formatted version of the Human Oral Microbiome Database (HOMD) [6]. Non-parametric Wilcoxon-Mann-Whitney tests were performed in R in order to verify if there were significant differences in terms of OTU abundances between the matrices obtained with the two databases. The correlation analysis was carried out using the psych package in R environment to identify patterns of co-occurrence/exclusion between OTUs or between OTUs and metabolites. Multiple-testing corrected pairwise Spearman correlations were computed between OTUs at the genus level (abundance >0.1% in at least 5 samples) and metabolites or between OTUs (abundance >0.01% in at least 5 samples). Co-occurrence/exclusion matrices were plotted using the corrplot package in R. Only significant correlations (False Discovery Rate ‘FDR’<0.05) were considered.

Samples were clustered using the Jensen–Shannon distance and partitioning around medoid (PAM) clustering; the optimal number of clusters was estimated using the Calinski–Harabasz (CH) index, and the silhouette validation technique was used for assessing the robustness of clusters, as previously described [7]. The analysis was carried out in the R environment using the cluster and ade4 packages.

Weighted and unweighted UniFrac distance matrices and OTU tables were used to perform ADONIS and ANOSIM statistical tests through the compare_category.py script of QIIME to verify the influence of diet, sex, smoking, BMI and geographic origin on the microbial population and to verify whether the samples grouped into clusters were significantly different.

The core microbiome was defined by the OTUs occurring in 98% of the individuals.

**^1^H Nuclear magnetic resonance (NMR) spectroscopy analysis**

One ml of thawed sample was centrifuged for 15 min 14000 × g at 4°C. The supernatant (800 µl) was thoroughly homogenized by vortex-mixing with 80 ml of cold 100mM phosphate buffer in deuterium oxide (D_2_O), containing 10mM 3-trimethylsilyl-propanoic-2,2,3,3-d4 acid sodium salt (TSP), as an internal standard. After adjusting the pH to 7.00, samples were centrifuged at 14000 × g for 5 min in order to further remove particulate. All ^1^H-NMR spectra were recorded at 300 K on a Bruker US+ Avance III spectrometer operating at 600 MHz, equipped with a BBI-z probe and a B-ACS 60 sampler for automation (Bruker BioSpin, Karlsruhe, Germany). The spectra were collected with a 90° pulse of 14 µs with 10W of power, a relaxation delay of 7 sec, and an acquisition time of 2.28 sec. Spectra were registered by means of the first increment of the nuclear overhauser effect spectroscopy pulse sequence [8]. This sequence is designed to suppress the water residual signal, while giving for each kind of proton of each substance peaks proportional to the concentration of the substance itself. Spectra were obtained by collecting 256 scans into 32 K data points covering a 20 ppm spectral width. Phase and baseline corrections were automatically performed using TopSpin version 3.0 (Bruker BioSpin). The chemical shifts were internally referenced to the TSP signal. The spectra were corrected for errors in chemical shift misalignments using an interval correlation optimized shifting procedure [9]. Signals assignment was carried out on the basis of literature [10, 11] and by using Amix software (version 2.1.3, Bruker BioSpin). The spectra were finally averaged over portions of 0.018 ppm.

**Gas-chromatography mass spectrometry-solid-phase microextraction (GC-MS/SPME) analysis of salivary volatile compounds**

After preconditioning, a carboxen/polydimethylsiloxane (CAR/PDMS) fiber (85 μm) and a manual solid phase micro-extraction (SPME) holder (Supelco Inc., Bellefonte, PA, USA) were used according to the manufacturer’s instructions. Before headspace sampling, the fiber was exposed to GC inlet for 10 min for thermal desorption at 250°C. Three grams of salivary sample were placed into 10 ml glass vials and added with 10 μl of 4-methyl-2-pentanol (final concentration of 33 mg/l), as the internal standard. Samples were then equilibrated for 10 min at 50°C. SPME fiber was exposed to each sample for 40 min. Both equilibration and absorption phases were carried out with stirring. The fiber was then inserted into the injection port of the gas chromatograph for 10 min for sample desorption. GC-MS analyses were carried out with an Agilent 7890A gas chromatograph (Agilent Technologies, Palo Alto, CA) coupled with an Agilent 5975C mass selective detector operating in an electron impact mode (ionization voltage, 70 eV). A Varian CP 7773 Wax 52 CB capillary column (length, 50 m; inside diameter, 0.32 mm x 1.2 μm; Agilent Technologies) was used. The temperature program was 40°C for 1 min, followed by an increase, at a rate of 4.5°C/min, to 65°C, an increase, at a rate of 10°C/min, to 230°C, and then 230°C for 17 min. The injector, interface and ion source temperatures were 250, 250, and 230°C, respectively. The mass-to-charge ratio interval was 30 to 550 Da at a rate of 2.9 scans per sec. Injection was carried out in splitless mode, and helium (flow rate, 1 ml/min) was used as the carrier gas. Molecules were identified based on comparison of their retention times with those of pure compounds (Sigma-Aldrich, Milan, Italy). Identities were confirmed by searching mass spectra in the available databases (NIST, version 2005; Wiley, version 1996). All the GC-MS raw files were converted to netCDF format via Chemstation (Agilent Technologies) and subsequently processed with the XCMS toolbox (http://metlin.scripps.edu/download/). XCMS software allows automatic and simultaneous retention time alignment, matched filtration, peak detection and peak matching. The resulting table containing information such as peak index (retention time-m/z pair) and peak area was exported into R (www.r-project.org) for subsequent statistical and multivariate analyses. Quantitative data for the identified compounds were obtained by the interpolation of the relative areas versus the internal standard area [12].

**Supplementary references**

1. Ercolini D, De Filippis F, La Storia A, Iacono M (2012) “Remake” by high-throughput sequemcing of the microbiota involved in the production of water buffalo Mozzarella cheese. Appl Environ Microbiol 78: 8142-8145.
2. Reeder J, Knight R (2010) Rapidly denoising pyrosequencing amplicon reads by exploiting rank-abundance distributions. Nat Methods 7: 668-669.
3. Edgar RC (2010) Search and clustering orders of magnitude faster than BLAST. Bioinformatics 26: 2460–2461.
4. Wang Q, Garrity GM, Tiedje JM, Cole JR (2007) Naȉve Bayesan classifier for rapid assignment of rRNA sequences into the new bacterial taxonomy. Appl Environ Microbiol 73: 5261–5267.
5. McDonald D, Price MN, Goodrich J, Nawrocki EP, De Santis TZ, et al. (2012) An improved Greengenes taxonomy with explicit ranks for ecological and evolutionary analyses of bacteria and archea. ISME J 6: 610-618.
6. Chen T, Yu WH, Izard J, Baranova OB, Lackshmanan A, et al. (2010) The Human Oral Microbiome Database: a web accessible resource for investigating oral microbe taxonomic and genomic information. Database. doi: 10.1093/database/baq013.
7. Arumugam M, Raes J, Pelletier R, Le Paslier D, Yamada T, et al. (2011) Enterotypes of the human gut microbiome. Nature 473: 174-180.
8. Alum MF, Shaw PA, Sweatman BC, Ubhi BK, Haselden JN, et al. (2008) 4,4-dimethyl-4-silapentane-1-ammonium trifluoroacetate (DSA), a promising universal internal standard for NMR-based metabolomic profiling studies in biofluids, including blood, plasma and serum. Metabolomics 4: 122–127.
9. Savorani F, Tomasi G, Engelsen SB (2010) Icoshift: a versatile tool for the rapid alignment of 1D NMR spectra. J Magn Reson 202: 190–202.
10. Takeda I, Stretch C, Barnaby P, Bhatnager K, Rankin K, et al. (2009) Understanding the human salivary metabolome. NMR Biomed 22: 577-584.
11. Silwood CJL, Lynch EJ, Seddon S, Sheerin A, Claxon AW, et al. (1999) ^1^H-NMR analysis of microbial-derived organic acids in primary root carious lesions and saliva. NMR in Biomed. 12: 345-356.
12. De Angelis M, Piccolo M, Vannini L, Siragusa S, De Giacomo A, et al. (2013) Fecal microbiota and metabolome of children with autism and pervasive developmental disorder not otherwise specified. PLoS One 8: e76993.
